# Successful Development of Bacteriocins into Therapeutic Formulation for Treatment of MRSA Skin Infection in a Murine Model

**DOI:** 10.1128/AAC.00829-20

**Published:** 2020-11-17

**Authors:** Kirill V. Ovchinnikov, Christian Kranjec, Tage Thorstensen, Harald Carlsen, Dzung B. Diep

**Affiliations:** a Faculty of Chemistry, Biotechnology and Food Science, Norwegian University of Life Sciences, Ås, Norway; b Department of Plant Molecular Biology, Norwegian Institute of Bioeconomy Research, Ås, Norway

**Keywords:** bacteriocins, garvicin KS, micrococcin P1, MRSA, skin infection, murine model

## Abstract

The emergence of antibiotic-resistant pathogens has caused a serious worldwide problem in infection treatment in recent years. One of the pathogens is methicillin-resistant Staphylococcus aureus (MRSA), which is a major cause of skin and soft tissue infections. Alternative strategies and novel sources of antimicrobials to solve antibiotic resistance problems are urgently needed. In this study, we explored the potential of two broad-spectrum bacteriocins, garvicin KS and micrococcin P1, in skin infection treatments. The two bacteriocins acted synergistically with each other and with penicillin G in killing MRSA *in vitro*. The MICs of the antimicrobials in the three-component mixture were 40 ng/ml for micrococcin P1 and 2 μg/ml for garvicin KS and penicillin G, which were 62, 16, and at least 1,250 times lower than their MICs when assessed individually. To assess its therapeutic potential further, we challenged the three-component formulation in a murine skin infection model with the multidrug-resistant luciferase-tagged MRSA Xen31, a strain derived from the clinical isolate S. aureus ATCC 33591. Using the tagged-luciferase activity as a reporter for the presence of Xen31 in wounds, we demonstrated that the three-component formulation was efficient in eradicating the pathogen from treated wounds. Furthermore, compared to Fucidin cream, which is an antibiotic commonly used in skin infection treatments, our formulation was also superior in terms of preventing resistance development.

## Abstract (precis)

The emergence of antibiotic-resistant pathogens has caused a serious worldwide problem in infection treatment in recent years. One of the pathogens is methicillin-resistant Staphylococcus aureus (MRSA), which is a major cause of skin and soft tissue infections. Alternative strategies and novel sources of antimicrobials to solve antibiotic resistance problems are urgently needed. In this study, we explored the potential of two broad-spectrum bacteriocins, garvicin KS and micrococcin P1, in skin infection treatments.

## INTRODUCTION

The emergence of multidrug-resistant bacterial pathogens is currently considered a global public health problem (1, 2). Lately, the Centers for Disease Control and Prevention (CDC) declared that the human race is now in the “postantibiotic era,” and the World Health Organization (WHO) warned that the antibiotic resistance crisis is becoming dire (3). Staphylococcus aureus is both a frequent commensal and one of the most important pathogenic microorganisms causing hospital- and community-acquired infections (4). S. aureus can colonize the anterior nares, axillae, groin, and gastrointestinal tract, making a reservoir from which bacteria can start infection if host defenses are compromised (5). In clinical settings, the bacterium is the leading cause of endocarditis, bacteremia, and chronic osteomyelitis (6). Moreover, S. aureus strains are also among the most common causes of infections related to medical instrumentation, including central-line-associated bloodstream infections and surgical wound infections (7). Strains of S. aureus are also the major cause of skin and soft tissue infections (SSTIs), and the global spread of methicillin-resistant S. aureus (MRSA) has turned the infection of simple cuts into a potential cause of serious infections, particularly in severely ill hospitalized patients (i.e., in long‐term care facilities) (8, 9).

The first observation of MRSA strains among clinical isolates was made as early as 1961, just 2 years after the introduction of methicillin to treat penicillin-resistant S. aureus infections; however, only since the 1990s have community-associated MRSA (CA-MRSA) strains spread in the general population worldwide (6). Since MRSA strains in hospitals are highly resistant to many other antibiotics, patients with SSTIs caused by MRSA have higher risk of bacteremia, hospital readmission, and death and require longer and more expensive periods of hospitalization than patients infected with other *Staphylococcus* species (10).

The current treatment regimen for patients hospitalized with MRSA-associated SSTIs includes vancomycin, linezolid, daptomycin, or ceftaroline (11); however, S. aureus clinical isolates resistant to these antibiotics have emerged within the past 20 years (12–15).

Thus, there is an urgent need for new antimicrobial agents with different bactericidal mechanisms fostering alternative therapeutic strategies to counteract MRSA infections.

Bacteriocins are ribosomally synthesized antimicrobial peptides that are generally stable over broad ranges of temperature and pH, with low toxicity and high antimicrobial activity at therapeutically acceptable concentrations (16, 17). Bacteriocins are produced by bacteria to kill other bacteria in order to compete for nutrients or habitats. Since bacteriocins have modes of action different from those of antibiotics, they are normally active against both antibiotic-sensitive and -resistant bacteria (18). Thus, bacteriocins represent a great source of antimicrobials that can be exploited to combat bacterial infections, especially against antibiotic-resistant pathogens.

Garvicin KS (GarKS) and micrococcin P1 (MP1) are two such bacteriocins with great therapeutic potential, as they are potent against a wide range of pathogens, including many Gram-positive bacteria, such as S. aureus, Enterococcus faecalis, Enterococcus faecium, *Listeria* spp., and *Streptococcus* spp. (19, 20). In addition, they are built differently and have different modes of action. GarKS is a multipeptide bacteriocin composed of 3 nonmodified peptides and belongs to the leaderless family (20). It kills cells by disrupting membrane integrity, leading to leakage of extracellular fluids over the membrane with subsequent lysis and cell death (unpublished data). MP1 is a heavily modified peptide that kills target cells by inhibiting protein synthesis (21).

In this study, we aimed to explore the therapeutic potential of GarKS and MP1. A search for synergies between these two bacteriocins and other antimicrobials was performed in order to achieve higher potency and, more importantly, to prevent resistance development. To evaluate their therapeutic potential, we developed a murine skin excisional-wound model to propagate S. aureus MRSA infections *in vivo*. Further, using a luciferase-tagged MRSA strain, we studied infection and treatment in live animals; this approach provided us an excellent tool to follow the dynamics of infection and treatment during the course of the experiments. Here, we report the successful development of bacteriocins into a drug formulation for therapeutic use against MRSA infection in a murine model.

## RESULTS

### Evaluation of synergetic antimicrobial activities.

**FIG 1:**
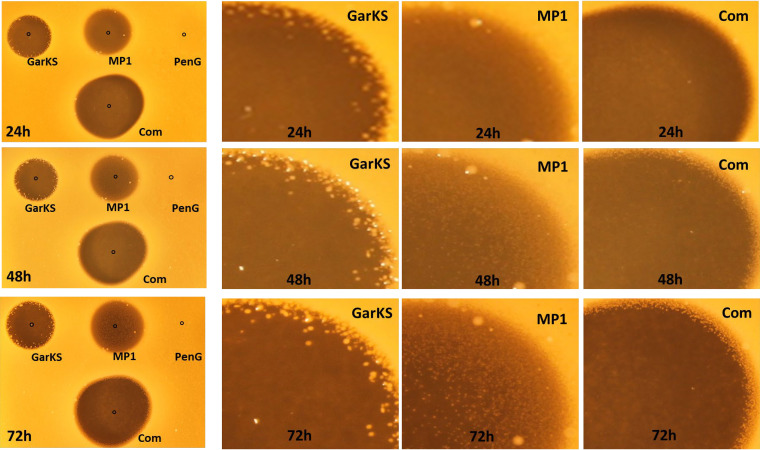
Assessment of the antimicrobials GarKS, MP1, and PenG, individually and in combination (Com), against MRSA ATCC 33521-lux. (Left column) Pure GarKS and PenG, each at 50 μg, and MP1 at 0.1 μg were applied at the indicated spots. The images of the plates were taken after 24, 48, and 72 h of incubation at 37°C. (Right three columns) Sections of the inhibition zones showing resistance development, seen as small white dots in the inhibition zones of GarKS and MP1.

GarKS and MP1 are two broad-spectrum bacteriocins whose MIC values vary depending on the types of indicators used. For instance, GarKS MIC values against lactococci, listeria, and enterococci are in the range of 1 to 20 μg/ml, while those of MP1 are in the range of 1 to 10 μg/ml (see Table S1 in the supplemental material). MIC values against S. aureus are 32 μg/ml for GarKS and 2.5 μg/ml for MP1 (Table 1). However, it is well known that antimicrobials often become ineffective in single-antimicrobial formulations. This is also true for GarKS and MP1, as can be seen in Fig. 1 with an MRSA strain—with long incubation, resistant colonies of MRSA appeared within the inhibition zones where single antimicrobials were applied. Using a checkerboard assay, some synergistic effect was seen between GarKS and MP1. Their MIC values were reduced to 0.16 μg/ml for MP1 and 8 μg/ml for GarKS when combined (Table 1). The fractional inhibition concentration (FIC) for the GarKS and MP1 combination was 0.314 (FIC values of ≤0.5 are considered synergetic between two components).

**TABLE 1:** MIC values of different antimicrobials against three MRSA strains, assessed individually and in combination with bacteriocins

Antimicrobial	MIC (μg/ml) for strain:							
	MRSA ATCC 33591-lux		MRSA USA-300		MRSA 43484		All three strains^*a*^	
	Single antimicrobial	Combined with GarKS (antibiotic/GarKS)	Single antimicrobial	Combined with GarKS (antibiotic/GarKS)	Single antimicrobial	Combined with GarKS (antibiotic/GarKS)	Single antimicrobial	Combined with GarKS (antibiotic/GarKS)
GarKS	32		32		32		32	
MP1	2.5	0.16/8	2.5	0.16/8	2.5	0.16/8	2.5	0.16/8
Amp	>2,500	16/8	>2,500	16/8	>2,500	16/8	>2,500	16/8
PenG	>2,500	16/8	>2,500	16/8	>2,500	16/8	>2,500	16/8
Kan	>125	32/32	>125	32/32	>125	32/32	>125	32/32
Ery	>250	62/32	31	16/8	62	32/16	31 to >2,500	16 to 62/8 to 32
Str	>2,500	32/8	62	32/16	62	32/16	62 to >2,500	32/8 to 16
Tet	150	38/32	1.2	0.6/16	1.2	0.6/16	1.2 to 150	0.6 to 37/16 to 32
Cam	125	32/32	125	64/32	16	64/32	16 to 125	32 to 64/8 to 32
								
		**Combined with MP1 (antibiotic/MP1)**		**Combined with MP1 (antibiotic/MP1)**		**Combined with MP1 (antibiotic/MP1)**		**Combined with MP1 (antibiotic/MP1)**
PenG		16/0.16		32/0.3		32/0.3		16 to 32/0.16 to 0.3
								
		**Combined with GarKS and MP1 (antibiotic/GarKS/MP1)**		**Combined with GarKS and MP1 (antibiotic/GarKS/MP1)**		**Combined with GarKS and MP1 (antibiotic/GarKS/MP1)**		**Combined with GarKS and MP1 (antibiotic/GarKS/MP1)**
PenG		8/4/0.08		8/4/0.08		8/4/0.08		8/4/0.08
								

a Summary of the MIC values against all three strains.

In order to seek stronger synergy, we assessed the synergistic effect between GarKS and a selected set of common and well-characterized antibiotics (Table 1). The antibiotics had very poor activity against at least one of the three MRSA strains (ATCC 33591-lux, USA-300, and MRSA 43484) tested. For instance, all three MRSA strains were cross-resistant to ampicillin and penicillin, as expected (MIC > 2,500 μg/ml). They were also resistant to kanamycin (MIC > 125 μg/ml). Furthermore, the MRSA strain ATCC 33591-lux was the most multiresistant, as it also tolerated higher concentrations of erythromycin (Ery) and streptomycin (Str) (MIC > 250 μg/ml and > 2,500 μg/ml, respectively).

Using a checkerboard assay against the three MRSA strains, we found no synergy or only additive effects between GarKS and kanamycin (Kan), Ery, Str, tetracycline (Tet), or chloramphenicol (Cam). Str showed moderate synergy with GarKS against only two MRSA strains out of three. At the same time, moderate synergy on a similar scale was observed between GarKS, ampicillin (Amp), and penicillin G (PenG) toward all three MRSA strains (Table 1). When combined, the MIC value of Amp or PenG was reduced at least 150 times (from over 2,500 μg/ml to 16 μg/ml), while that of GarKS was reduced 4 times (from 32 μg/ml to 8 μg/ml). The FIC value for the GarKS and PenG combination was 0.26. PenG is a relatively low-cost product compared to Amp (22) and is also still the drug of choice for treatment of S. pyogenes, another common cause of SSTI (23). PenG was therefore chosen for further synergy assessment with MP1.

By a checkerboard assay, we found great synergy of MP1 with PenG: the MIC value of PenG was reduced 80 to 150 times (from 2,500 μg/ml to 16 to 32 μg/ml), while the MIC of MP1 was reduced 8 to 16 times (from 2.5 μg/ml to 0.16 to 0.3 μg/ml) (Table 1). The FIC value for the MP1 and PenG combination was 0.07 to 0.13, depending on the MRSA strain.

As PenG, GarKS, and MP1 appear to have synergy in dual combinations, we also assessed whether we could improve the synergy further by combining all three. Indeed, stronger synergy was found in the triple combination: the MIC of PenG was reduced more than 300 times (from 2,500 μg/ml to 8 μg/ml), that of MP1 about 30 times (2.5 μg/ml to 0.08 μg/ml), and that of garvicin KS about 8 times (from 32 μg/ml to 4 μg/ml) (Table 1). Combining the three antimicrobials also prevented development of MRSA-resistant colonies on agar plates, at least after 72 h of incubation at 37°C (Fig. 1). The FIC value for the three-component formulation was 0.16, confirming strong synergy between the antimicrobials.

In addition, we tested a larger panel of S. aureus strains (see Table S2 in the supplemental material) and two other typical skin infection pathogens, Staphylococcus pseudintermedius and E. faecalis (see Fig. S1 in the supplemental material). As expected, the triple combination was active against all the strains tested, including those resistant to other antibiotics.

### Choosing the vector for the formulation.

Before testing the therapeutic properties of the triple combination in a murine model (see below), we searched for a suitable solvent (referred to here as the vector) to carry the three antimicrobials. Since the GarKS peptides, and especially MP1, are relatively hydrophobic molecules, it was important to look for vectors that could dissolve these peptides, as well as at the same time allowing us to assess antimicrobial activity. GarKS, PenG, and MP1 dissolved well in white Vaseline, but the Vaseline mixture showed poor antimicrobial activity on agar plate assays (data not shown); this negative result was probably due to strong hydrophobic interactions between the molecules and the Vaseline, preventing diffusion of the antimicrobials.

Next, we tested hydroxypropyl cellulose (HPC), which is a more hydrophilic polymer commonly used for topical and oral pharmaceutical preparations (24). The three antimicrobials were less soluble in HPC, but, based on the solubility of the individual components, we were able to make a mixture containing 5 mg/ml GarKS, 5 mg/ml PenG, and 0.1 mg/ml MP1 in 5% HPC that was easy to handle. The mixture was relatively stable, as it did not lose its activity after storage at 5°C for 1 month (data not shown). Interestingly, when GarKS, MP1, and PenG were dissolved in 5% HPC, the mixture in HPC increased the activity 2-fold, with final MICs for GarKS and PenG at 2 μg/ml and 40 ng/ml for MP1. The mixture containing 5 mg/ml GarKS, 5 mg/ml PenG, and 0.1 mg/ml MP1 in 5% HPC is called “the formulation” here.

### Comparison of our formulation with the commercial Fucidin cream *in vitro*.

**FIG 2:**
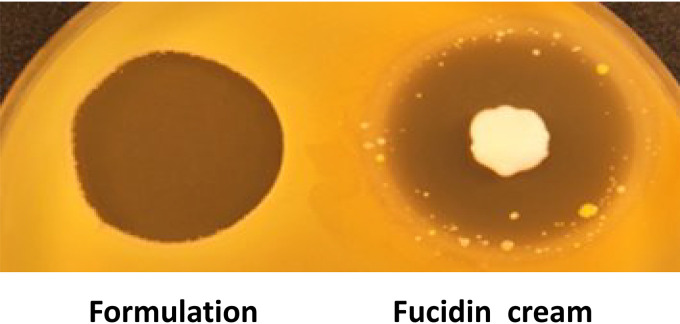
Comparison of the antimicrobial activities of the three-component formulation and Fucidin cream (Leo Pharma, Denmark) against MRSA ATCC 33591-lux. Both antimicrobials were applied to the indicated spots (formulation with 250 μg GarKS, 250 μg PenG, and 5 μg MP1 and Fucidin cream containing 1 mg fusidic acid).

Once the formulation was created and its stability in HPC was confirmed, it was imperative to compare it with available commercial skin antimicrobial products. We decided to use Fucidin cream (Leo Pharma, Denmark), since it is widely used as a topical agent against bacterial skin infections, with fusidic acid as the active ingredient (25). As can be seen in Fig. 2, in terms of antimicrobial activity toward MRSA, our formulation appeared comparable or superior to Fucidin cream. It is noteworthy that resistant colonies appeared within the inhibition zone of Fucidin, while no such resistance development was seen with the formulation.

### The formulation effectively inhibits the growth of MRSA in a murine skin infection model.

**FIG 3:**
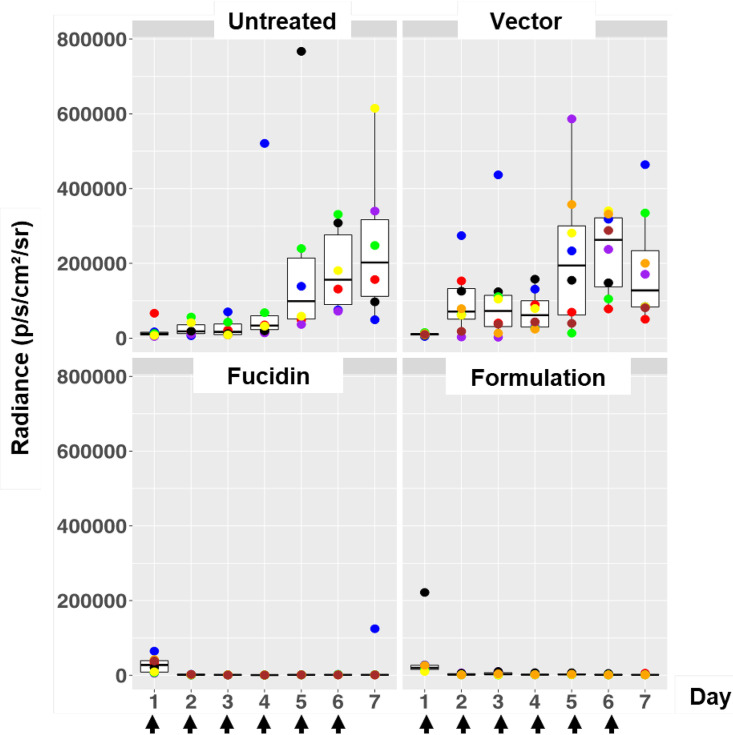
Box plots of bioluminescent signal produced by MRSA ATCC 33591-lux in photons per second per square centimeter per steradian in differently treated mouse groups. The days of treatment with the formulation, Fucidin cream, and vector are indicated with arrows. Untreated mice were infected and left untreated. The area within each box represents the interquartile region (IQR), which comprises the second and third quartiles and describes the interval of values where the middle 50% of the observed data are distributed. The horizontal black line within each box represents the median value. The extent of the IQR (box height) express the degree of variability measured within the middle 50% of the observed data, with whiskers extending out at either side of the boxes marking the minimum and maximum observed values, as well as the variability outside the middle 50% of values (whisker length). Outliers are displayed as data that extend out of the whisker limit (1.5 times the IQR). The numbers of mice were 6 in the untreated group, 8 in the vector group, 8 in the Fucidin group, and 7 in the formulation group.

**FIG 4:**
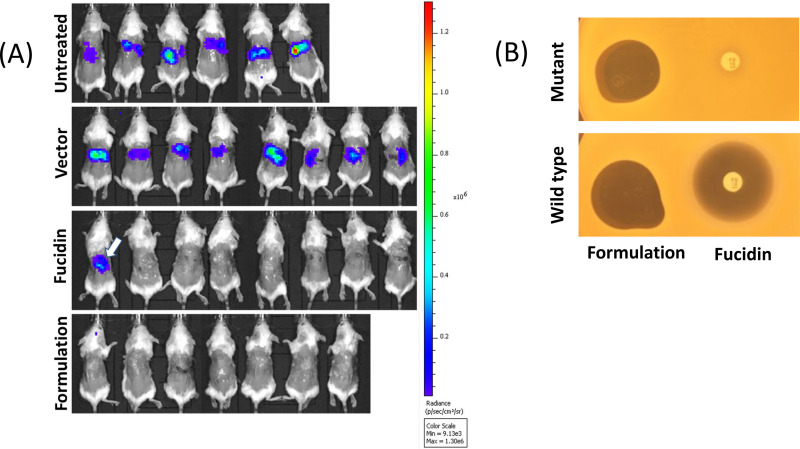
(A) *In vivo* imaging of bioluminescent signal produced by MRSA ATCC 33591-lux in photons per second per square centimeter per steradian from the different mouse groups on the last day of the experiment (7 days postinfection, after six treatments). The arrow in the Fucidin group image indicates a Fucidin-resistant mutant population appearing on the mouse after 6 consecutive days of treatments (Fig. 3). (B) Fucidin resistance develops during the treatment of mice. Bacterial cells isolated from the wound with strong bioluminescent signal in the Fucidin-treated group (arrow in panel A) were rechallenged and shown to be resistant to a fusidic acid disc, but not to the formulation. Wild-type MRSA ATCC 33591-lux cells exposed to the formulation and Fucidin were sensitive to both antimicrobials.

In order to assess the therapeutic efficacy of our formulation *in vivo*, we developed a murine wound infection model. For this, a luciferase-expressing MRSA strain, S. aureus ATCC 33591-lux (PerkinElmer), was used to monitor the therapeutic effect of the formulation. Fucidin cream was again used as a positive control. In all the experiments, wounds on the back of each animal were made by skin punching followed by inoculation of the same amount of MRSA ATCC 33591-lux (Xen31) (2 × 10^7^/wound). The day when the inoculation was performed is referred to day 0. Treatment started on day 1, i.e., 24 h after infection/inoculation. Three consecutive experiments were performed to optimize the treatment regimen. In the first experiment, we examined whether the formulation had a good therapeutic effect on infected mice and whether multiple treatments might have an adverse effect on their behavior. We treated the wounds once a day for the entire period of 7 days. As expected, light emissions from luciferase activity from all the mice were high prior to the treatments (Fig. 3). Emissions from nontreated mice then increased strongly and more or less stabilized within 3 to 4 days. In both treated groups (formulation and Fucidin), on the other hand, the luciferase signal declined sharply the first day after treatment (day 2) to nearly undetectable levels and stayed low over the entire period. Interestingly, we noticed that the signal reappeared in one of the Fucidin-treated mice at day 7 (Fig. 4A), indicating possible resistance development. To confirm this, a luminescent isolate from the mouse wound was rechallenged with the antimicrobials. As expected, the isolate was indeed resistant to Fucidin, but not to the formulation (Fig. 4B).

All the mice showed no obvious signs of abnormal behavior, either in the nontreated group or in the treated groups (vector, Fucidin, and formulation), indicating that the formulation had no obvious toxic effects.

**FIG 5:**
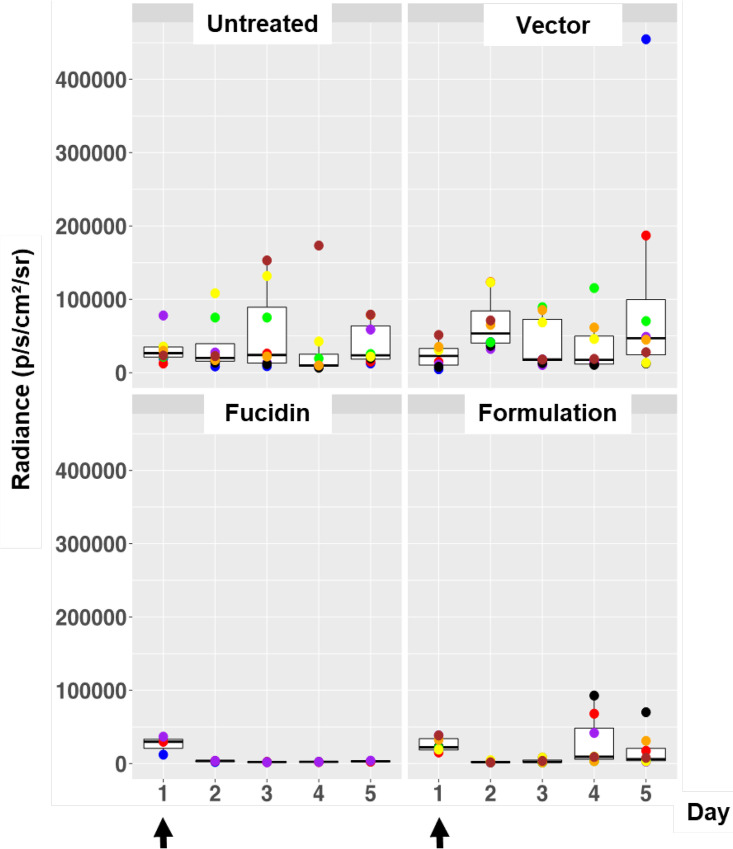
Box plots of bioluminescent signal produced by MRSA ATCC 33591-lux in photons per second per square centimeter per steradian in differently treated mouse groups. Application of the formulation, Fucidin creme, and vector (5% HPC) was done only once on day 1 postinfection (arrows). Untreated mice were infected and left untreated. The box plot description is the same as for Fig. 3. The number of mice was 8 in all groups, except for the Fucidin group, where it was 4.

Given the good therapeutic effect of the formulation, we next examined the lasting effect of one treatment. We performed infection as described above but with only one treatment (performed 24 h after infection), followed by daily imaging sessions with no additional treatment. As can be seen in Fig. 5, for the untreated and vector-treated groups, the signals stayed relatively high compared to the positive control (the Fucidin-treated group). As expected, the relatively high signals at the beginning disappeared quickly in both formulation- and Fucidin-treated mice at day 2, but the signal in the formulation group reappeared on day 4 and stayed elevated during the rest of the experiment. The Fucidin group remained low over the entire period (Fig. 5).

**FIG 6:**
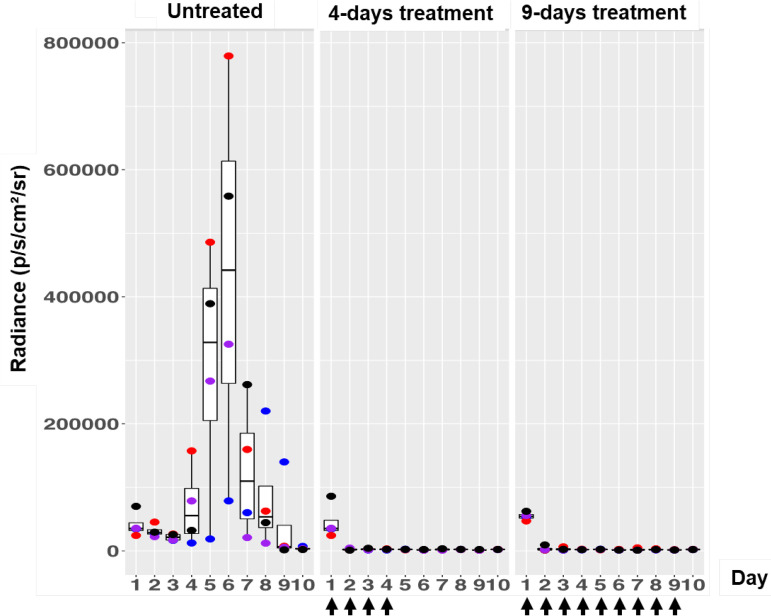
Box plots of bioluminescent signal produced by MRSA ATCC 33591-lux in photons per second per square centimeter per steradian in differently treated mouse groups. Application of the formulation was done only four times (4 days of treatment) or nine times (9 days of treatment). The days of treatment are indicated by arrows. The untreated group was infected but not treated. The box plot description is the same as for Fig. 3. The number of mice was 4 in each group.

Based on the above observations that one treatment was not sufficient to suppress the infection for a long period, we decided to increase the number of treatments with the formulation to four times, i.e., once a day during the first 4 days after the infection day. Again, we scored the emission signal over the entire period of the experiment (10 days). For comparison, we also included two control groups: one untreated and one treated with formulation every day over the entire experiment (except day 10 to score only the light signals). As seen in Fig. 6, the formulation again caused a sharp decline in luciferase signal after treatment, and it stayed low for at least 6 days after the last treatment on day 4. As expected, the same was true for the group treated daily for 9 days with the formulation. We thus concluded that a regimen of 4 days of treatment once a day was enough to create a long-lasting antibacterial effect of the formulation.

## DISCUSSION

In this study, we searched for a combination of antimicrobials that has strong synergy against MRSA. Two of the antimicrobials we used were bacteriocins: GarKS, which has been recently discovered and displays broad-spectrum activity (20), and MP1, which has been known for decades for its great potential in therapeutic treatments, especially against Gram-positive pathogens (26). However, due to its strong hydrophobicity, MP1 has mostly been explored for its potential in topical treatments (27). The last antimicrobial was PenG, an antibiotic that has been of great importance in human medicine since World War II, but its heyday has long been over due to the rise of resistance development. When used in the formulation, the MIC values of the antimicrobials were 2 μg/ml for GarKS and PenG and 40 ng/ml for MP1, which are comparable to those of the most potent antibiotics used for the treatment of S. aureus infections. According to a EUCAST clinical-breakpoint review, S. aureus strains are classified as resistant if their MIC values are more than 1 μg/ml for fusidic acid and daptomycin, 2 μg/ml for vancomycin and teicoplanin, and 4 μg/ml for linezolid (https://www.eucast.org/clinical_breakpoints).

We found that a combination of the three antimicrobials was effective not only against MRSA, but also against many other Gram-positive pathogens common in skin infections, including coagulase-negative staphylococci and E. faecalis (see Fig. S1), thus demonstrating its broad therapeutic value in skin infection treatment.

The three antimicrobials described above individually do not have strong potency and/or long-lasting effects on MRSA. MP1 inhibits translation in bacteria by selective binding to a GTPase-associated center from which it prevents the elongation/translocation process (28). The ribosomal protein L11 is the target of MP1, but mutations in the gene encoding L11 quickly render cells resistant to MP1 (29). GarKS is very potent against many Gram-positive pathogens, including listeria, enterococci, streptococci, and bacilli, but has moderate potency against S. aureus, with MIC values around 30 μg/ml. The receptor for garvicin KS has not yet been clearly identified. Nonetheless, resistant mutants of Lactococcus lactis (with 4 to 8 times less sensitivity) have been isolated, and they all have mutations within *pspC* (data not shown), a gene believed to be involved in stress response in bacteria (30). Whether PspC is acting as a receptor for the bacteriocin or is involved in a process leading to resistance remains to be investigated. PenG and other lactams inhibit the growth of bacteria by binding to penicillin binding protein (PBP) to prevent cell wall synthesis (31). The major mechanism of resistance in MRSA is mediated by *mecA*, a gene encoding a protein called PBP-2a, which can replace the function of the common PBPs. PBP-2a has lower affinity for beta-lactams and can therefore allow cells to grow in the presence of the antibiotics (31). It was therefore surprising to see that our formulation sensitized MRSA strains to PenG, which was otherwise ineffective against MRSA when used alone. One might speculate whether the formulation triggers inactivation of PBP-2a by an unknown mechanism at the protein level or at the gene regulation level and that this inactivation renders MRSA sensitive to PenG again. Unraveling the mechanism underlying PenG resensitization is of great importance, as it will revitalize PenG and other beta-lactam antibiotics, a group of antibiotics that have had great success in human and animal medicine. However, such studies are beyond the scope of the present work and therefore more suitable to be addressed separately in the future.

We used HPC as a vector to carry the antimicrobials in a mixture for topical treatment of skin infection in a murine model. HPC is a synthesized cellulose derivative to which a hydroxypropyl group is introduced as a substitute for the 2,3,6-OH group, thereby rendering it soluble in both water and organic solvents. The amphiphilic property of HPC is important to dissolve the antimicrobials, especially with MP1, which is relatively hydrophobic, while GarKS peptides and PenG are more amphiphilic. HPC is known to be used in pharmaceuticals as a viscosity, coating, or drug release-modifying agent (32). Cellulose derivatives have been used for formulating hydrophobic antimicrobials (33) and successfully used *in vivo* in a mouse skin wound model for slow release of antimicrobial peptide, PXL150 (34). Using 5% HPC, we were able to dissolve 5 mg/ml GarKS, 5 mg/ml PenG, and 0.1 mg/ml MP1 as a synergistic mixture effective against MRSA both on agar plates and in the skin wound model. To compare it with some commercial drugs common in skin treatment, Fucidin cream has 20 mg/ml of fusidic acid (Leo Pharma, Denmark), while PenG is used in the range of 30 to 60 mg/ml (Mastipen Vet; VetPharma, Norway) and chlorhexidine acetate at 5 mg/g (bacimycin; Actavis Group). In our case, the concentrations of the antimicrobials in our mixture were limited by their solubility in the HPC solution.

In many studies the time between inoculation of the pathogen on skin wounds and the first treatment is rather short, 5 to 30 min (35–37), while in others it was in 1 to 4 h (38–41). Our model was much more realistic and conservative, as we allowed the pathogen to grow for 24 h to initiate and establish infection before the first treatment took place. Further, we used a strain derived from MRSA ATCC 33591 in our infection model; the strain is known to produce most of its biofilm on surfaces within the first 24 h after inoculation (42). Thus, this 24-h gap between infecting the mice and the first treatment is close to clinical settings, where biofilm is commonly associated with skin infection, increasing resistance to antibiotics. This fact has previously been shown with MRSA ATCC 33591, where the 24-h biofilm-associated cells were very resistant to antibiotics compared to 6-h biofilm-associated or planktonic cells (43).

In the first mouse experiment, we treated the wound with the formulation every day and demonstrated that this treatment indeed caused a strong reduction in the bacterial load as judged from the absent or very low luciferase signals from the wounds. This shows that the doses used in the formulation reached a sufficiently high concentration to kill bacteria in a realistic *in vivo* infection model. We further showed that a single treatment 1 day after inoculation initially caused a dramatic reduction of the pathogens in the wounds, but evidently a significant proportion of the pathogens survived and multiplied when the effect of treatment subsided. When we treated the mice for four consecutive days, we found no light emission coming from the wounds for at least 6 days after the end of treatment. Based on these experiments, we can conclude that more than one treatment is necessary to cause a long-lasting effect. These results were in contrast to those for Fucidin treatment, which caused a long-lasting effect even with only one treatment. The difference was likely due to the fact that our formulation was not 100% optimized for this protocol, and we suggest that components may have been released too fast after the treatment and may have been diluted before all the bacteria in the wound were killed. In contrast, the Fucidin used in the study was optimized for skin treatments and was most likely very efficient for this purpose. However, we discovered one potential caveat with Fucidin: when treated for seven consecutive days, one of the mice in the Fucidin group reestablished bacterial growth, which was caused by the development of Fucidin-resistant MRSA cells in the wound. This was confirmed in bacterial isolates from the mouse wound. Interestingly, the Fucidin-resistant isolate was still sensitive to the formulation (Fig. 4B). This result is also in line with the *in vitro* assay on agar plates, where Fucidin-resistant colonies easily developed within the inhibition zone of Fucidin while no obvious colonies appeared within the inhibition zone of our formulation (Fig. 2).

An important aspect worth mentioning is the toxicity of the formulation. How the formulation affected the physiology of the treated mice was not assessed in this study. However, based on the visual observation, no obvious signs of abnormal behavior were seen in mice treated daily compared to nontreated mice, indicating that toxicity was low or absent when mice were treated with the formulation in the settings applied.

Drug development is a long, complicated, multidisciplinary process that includes a large set of pharmaceutical challenges (stability, toxicity, delivery, pharmacokinetics, etc.) and a series of preclinical and clinical tests before a drug reaches the market. Moreover, drug development is also very costly and high risk, as novel antimicrobials can quickly become useless due to resistance development. Consequently, drug industries nowadays are quite reluctant to invest large amounts of money in developing novel antimicrobials unless they see benefits in a longer perspective. Our strategy has shown that, with the right bacteriocins and in the right combinations with other antimicrobials, they can be developed into efficient drug formulations that not only are highly potent against pathogens but also prevent resistance development. As most bacteriocins have different modes of action than traditional antibiotics, they have great potential as the next-generation antimicrobials, or at least as important supplements to our antimicrobial arsenal to fight antibiotic-resistant pathogens. Bacteriocins are ubiquitous in nature; they are very diverse in terms of chemistry, specificity, mode of action, etc.; and they have been widely exploited in food preservation for centuries but are still barely exploited in human medicine. Given the promising results presented in this work, this untapped source of antimicrobials should be exploited better for human medicine in the future.

## MATERIALS AND METHODS

### Bacterial strains and growth conditions.

*Staphylococcus* and *Enterococcus* strains were grown in brain heart infusion (BHI) broth (Oxoid, United Kingdom) at 37°C under aerobic conditions without shaking. For *in vivo* imaging of bacterial infection in mice, S. aureus Xen31 (Perkin Elmer, Waltham, MA) was used. The strain was derived from the parental strain S. aureus ATCC 33591, a clinical MRSA strain isolated from Elmhurst Hospital in New York, NY, USA (44). S. aureus Xen31 possesses a stable copy of the modified Photorhabdus luminescens *luxABCDE* operon at a single integration site on the bacterial chromosome. Other strains were taken from our collection (Laboratory of Microbial Gene Technology, Norwegian University of Life Sciences).

### Antimicrobial agents and formulation vector.

GarKS peptides were synthesized by Pepmic Co., Ltd. (China) with 90 to 99% purity and solubilized to concentrations of 1 to 10 mg/ml in Milli-Q water. MP1 was purchased from Cayman Chemical (United States) with ≥95% purity and stored at a concentration of 20 mg/ml in dimethyl sulfoxide. Antibiotics were obtained from Sigma-Aldrich (Norway) and solubilized to concentrations of 5 to 100 mg/ml according to the supplier’s instructions. All antimicrobials were stored at −20°C until they were used. The final formulation for mouse treatment was prepared in 5% (wt/vol) HPC with a weight-average molecular weight (*M*_w_) of ∼80,000 g/mol and a number-average molecular weight (*M*_n_) of ∼10,000 g/mol (Sigma-Aldrich, Norway).

### AAMP assay.

Antimicrobial activity was measured using a antimicrobial activity microtiter plate (AAMP) assay (45). The MIC is defined as the lowest concentration of an antimicrobial or antimicrobial combination that inhibits the visible growth (at least 50% growth inhibition) of a microorganism after 24-h incubation at 37°C in microtiter plates in 200 μl culture.

### Synergy assessment.

Synergy testing was done with a microtiter plate checkerboard assay as previously described (46). Briefly, equal amounts of antimicrobial A were applied to microtiter plate 1 in wells A1 to H1 and then diluted 2-fold into wells 2 to 12. Similarly, equal amounts of antimicrobial B were applied to microtiter plate 2 in wells A1 to A12 and diluted 2-fold into wells B to H. Volumes of 50 μl of antimicrobial A from each well of microtiter plate 1 were transferred into microtiter plate 3, except for wells H1 to H12. Similarly, the same volumes were transferred from microtiter plate 2 into plate 3, except for wells A1 to A8. A 100-μl volume of a 25-times-diluted overnight bacterial culture of MRSA was transferred into each well of plate 3. A 50-μl volume of pure broth was added to each well in lines H1 to H12 and A1 to A8, which were used to estimate the MIC values of pure substances (blank).

Three-component synergy was assessed by mixing the three components (100 μg of PenG, 50 μg of GarKS, and 2.5 μg of MP1) in one well of a microtiter plate and comparing its activity with the activity of individual antimicrobials on the same plate.

The FIC, which was used to define synergy, was calculated as follows: FIC = FICa + FICb + FICc, where FICa is the MIC of A in combination divided by the MIC of A alone, FICb is the MIC of B in combination divided by the MIC of B alone, and FICc is the MIC of C in combination divided by the MIC of C alone. Effects were considered synergistic if the FIC was ≤0.5 for a two-component mixture and ≤0.75 for a three-components mixture (47, 48).

### Murine experiments.

Experiments on mice were approved by the Norwegian Food Safety Authority (Oslo, Norway), application no. 18/57926. In total, 72 female BALB/cJRj mice at 4 weeks of age were purchased from Janvier (Le Genest-Saint-Isle, France). Two to four mice were housed per cage during the whole experiment and maintained on a 12 h light/12 h dark cycle with *ad libitum* access to water and a regular chow diet (RM1; SDS Diet, Essex, United Kingdom). The mice were acclimatized in our mouse facilities for 2 weeks before the start of the experiments; hence, the age of the mice at the start of the experiments was 6 weeks.

Before infection and treatment, the mice were shaved as follows. The mice were anesthetized with a Zoletil Forte (Virbac, Carros, France), Rompun (Bayer, Oslo, Norway), and Fentadon (Eurovet Animal Health, Bladel, The Netherlands) cocktail (ZRF) (containing 3.3 mg Zoletyl Forte, 0.5 mg Rompun, and 2.6 μg fentanyl per ml 0.9% NaCl) by intraperitoneal injection (0.1 ml ZRF/10 g body weight) and shaved on the back and flanks with an electric razor. The remaining hair was removed with hair removal cream (Veet; Reckitt Benckiser, Slough, United Kingdom) according to the manufacturer’s instructions. The next day, the mice were again anesthetized with ZRF cocktail (0.1 ml/10 g body weight), and two skin wounds were made on the back of each mouse with a sterile biopsy punch 6 mm in diameter (dermal biopsy punch; Miltex Inc., Bethpage, NY). Prior to infection, overnight-grown S. aureus Xen31 cells were washed twice in sterile saline and then suspended in ice-cold phosphate-buffered saline (PBS) buffer. Each wound was inoculated with 10 μl of PBS containing ca. 2 × 10^7^ CFU of S. aureus Xen31 cells using a pipette tip. After bacterial application, the mice were kept on a warm pad for 10 to 15 min to dry the inoculum, and the wounds were then covered with a 4- by 5-cm Tegaderm film (3M Medical Products, St. Paul, MN, USA). The mice were then left for 24 h for the infection to establish. The day after (24 h postinfection [p.i.]), the mice were anesthetized with 2% isoflurane, and the luminescent signal was measured with an IVIS Lumina II, (Perkin Elmer; 2-min exposure time). The luminescent signal was quantified with Living Image software (Perkin Elmer) from regions of interest (ROI) around the wound and expressed as photons per second per square centimeter per steradian.

From that point, groups of mice were subjected to 4 different treatments, either daily or on a selected number of days depending on the type of regimen designed (see below). The 4 different treatments were as follows: one mouse was treated with the bacteriocin-based formulation (5 mg/ml GarKS, 0.1 mg/ml MP1, 5 mg/ml PenG in 5% HPC gel), one mouse was treated with formulation vehicle (5% HPC gel) as a negative control, one mouse was with treated with Fucidin cream (2% fusidic acid in a cream base; Leo Pharma, Denmark) as a positive control, and one mouse was left untreated. To optimize the treatments, three different regimens were performed consecutively: regimen 1 involved treatments daily from day 1 p.i. until termination at day 7 (*n* = 29); regimen 2 involved only one treatment on day 1 p.i., with the mouse left untreated until termination (day 7) (*n* = 27); and regimen 3 involved four treatments on days 1, 2, 3, and 4 p.i., with the mouse left untreated until termination (day 10) (*n* = 16). In all cases, when treated, 40 μl per wound of either antibacterial solution or control substance was injected under the Tegaderm using an insulin syringe (BD SafetyGlide; 29G needle). The bioluminescent signal produced by S. aureus Xen31 luciferase was recorded once per day before the treatments during the entire course of the experiments. At the end of each experiment, the mice were euthanized by cervical dislocation.

### Statistical analysis.

All *in vitro* assays were done three times. The data in the graphs represent the luminescent signal obtained from the analysis of individual mice. For statistical analyses and graphs, R Studio (version 1.0.15; https://rstudio.com/products/rstudio/download/) was used.

## Supplementary Material

Supplemental file 1
