# Supplementary material for: Successful Development of Bacteriocins into Therapeutic Formulation for Treatment of MRSA Skin Infection in a Murine Model
Source: Antimicrob Agents Chemother. 2020 Nov 17;64(12):e00829-20. doi: 10.1128/AAC.00829-20 (PMC7674055; doi:10.1128/AAC.00829-20)
Supplement: Supplemental file 1 [file AAC.00829-20-s0001.pdf]

Supplemental Table 1. Range of MIC values (µg/ml) of GarKS and MP1 against selected bacteria.

Strain ↓/ Antimicrobial →	GarKS	MP1
Lactococci (n=7)	1-4	1-5
Listeria (n=6)	1-4	1-5
Enterococci (n=7)	2-20	2.5-10

Supplemental Table 2. MIC values of selected antimicrobials (µg/ml), as single components and formulation mixture against different strains of *S. aureus*.

Strain → Antibiotic ↓	Ery	Str	Tet	Cam	Kan	GarKS	MP1	PenG	Combined activity PenG/GarKS/MP1
LMG3322	<2.5	62	<0.3	8	64	16	>1.8	<0.24	<0.24/ <0.12 /<0.002
LMG3255	<2.5	125	2.4	16	>125	32	>1.8	<0.24	<0.24/ <0.12 /<0.002
LMG3271	<2.5	125	2.4	16	64	32	>1.8	<0.24	<0.24/ <0.12 /<0.002
LMG3314	<2.5	62	1.2	8	64	32	0.4	<0.24	<0.24/ <0.12 /<0.002
LMG3328	<2.5	62	1.2	8	32	32	0.9	<0.24	<0.24/ <0.12 /<0.002
LMG3307	<2.5	62	0.6	8	64	32	0.9	<0.24	<0.24/ <0.12 /<0.002
LMG3242	<2.5	250	1.2	8	>125	32	0.4	0.24	<0.24/ <0.12 /<0.002
LMG3264	<2.5	>250	2.4	16	>125	32	>1.8	4	0.5/0.24/0.003
LMG3266	>2500	>125	1.2	32	>125	32	>1.8	64	8/4/ 0.05
LMG3258	<2.5	62	1.2	16	125	32	1.8	64	8/4/ 0.05
LMG3263	>2500	31	2.4	32	>125	16	1.8	125	8/4/ 0.05
LMG3265	>2500	31	2.4	32	64	16	1.8	125	8/4/ 0.05
LMG3026	<2.5	31	77	8	16	16	>1.8	>2500	4/2/0.025

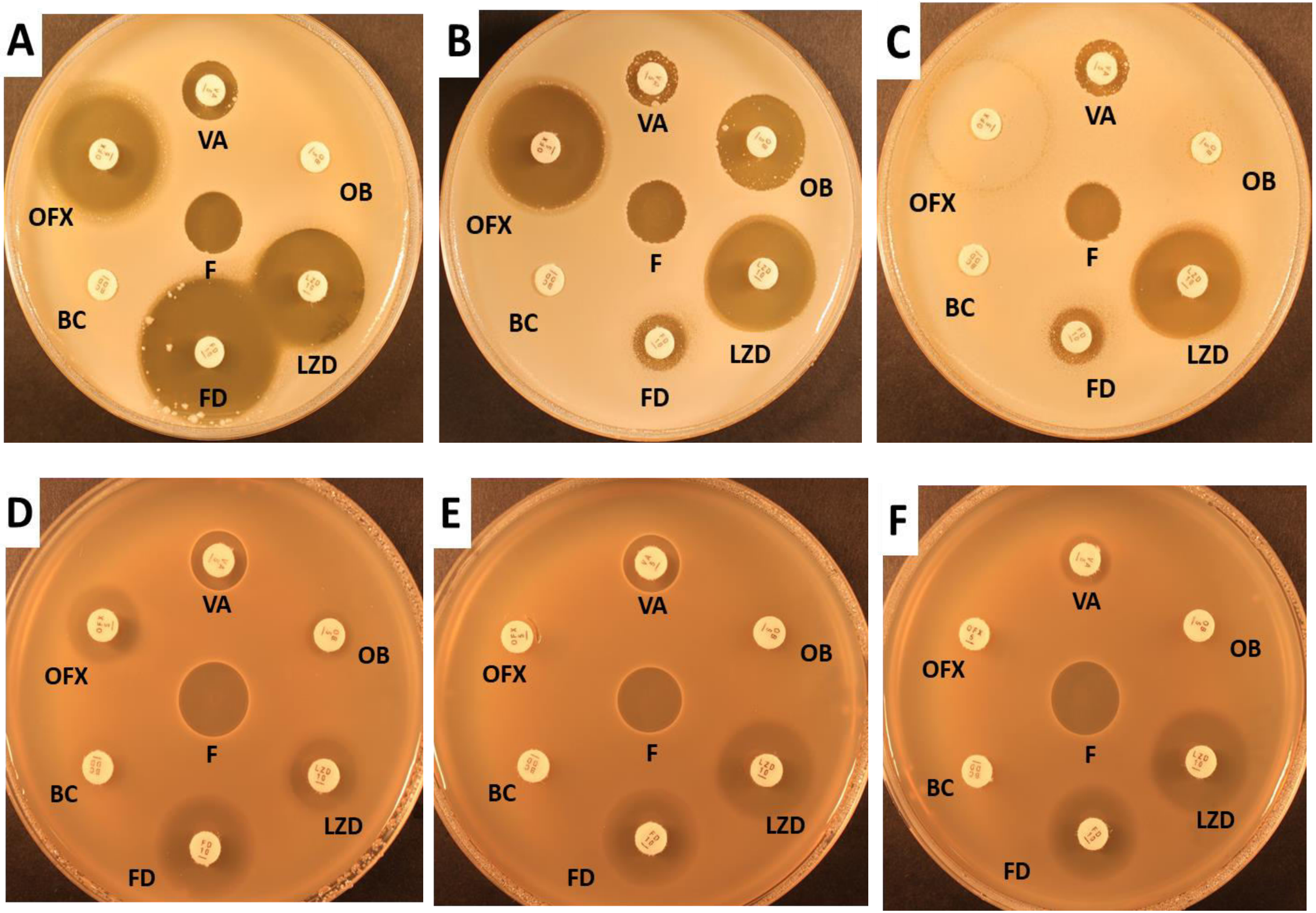

Supplemental Figure 1. Comparison of antimicrobial activity of the Formulation with selected antibiotics against S. pseudintermedius (A-C) and Enterococcus faecalis (D-F). Formulation (F) containing 15 µg for GarKS, 15 µg for PenG and 0.3 µg for MP1; Antibiotic discs – VA-vancomycin, 5 µg; OB – cloxacillin, 5 µg; LZD – linezolid, 10 µg; FD – fusidic acid, 10µg; BC – bacitracin, 0.04 Units; OFX – ofloxacin 5µg.

Supplemental Figure 1. Comparison of antimicrobial activity of the formulation with selected antibiotics against *S. pseudintermedius* (A-C) and *Enterococcus faecalis* (D-F). formulation (F) containing 15 µg for GarKS, 15 µg for PenG and 0.3 µg for MP1; Antibiotic discs – VA-vancomycin, 5 µg; OB – cloxacillin, 5 µg; LZD – linezolid, 10 µg; FD – fusidic acid, 10µg; BC – bacitracin, 0.04 Units; OFX – ofloxacin 5µg.
